# Editorial

**Published:** 1903-06-15

**Authors:** 


### EDITORIAL.

THE new dental law for the State of Illinois, after passing both branches of the legislature, was vetoed by the governor, and the indications are that it may not be taken up again. Just what the reasons may have been for the veto we are not advised, but the probabilities are that political rather than professional or civic influences are at the bottom of the matter. The lesson to be learned from such a downfall is to never move in an important matter with haste or without abundant resources. Things are done with such celerity in the vicinity of Chicago that it may not seem to those who had it in charge that this matter was not duly matured. But to us it seems that the bill had some weak points that should have been eliminated, and other features that should have been made stronger. Isn't it about time that our national organizations should take up this matter in earnest, and devise an ideal dental law which would be equitable and tend best to conserve the best interests of the several commonwealths of our nation?—at least, suggest the principles upon which all state legislation should be planned. It might not be possible to get universal acceptance; but combined wisdom expressed in this form would certainly have a great influence in modeling legislation and it would, so far as uniformity was secured, solve the vexed question of the transfer of practitioners from one State to another. This problem Dr. Ottolengui proposes to solve by a certificate of character issued by the Examining Board from the State a man leaves to the one in which he desires to practice.

